# Prolonged survival of patients with EGFR-mutated non-small cell lung cancer with solitary brain metastases treated with surgical resection of brain and lung lesions followed by EGFR TKIs

**DOI:** 10.1186/s12957-017-1252-y

**Published:** 2017-10-16

**Authors:** Qi Gui, Jiangang Liu, Dapeng Li, Chengcheng Xu

**Affiliations:** 1 Departments of Oncology, The First Affiliated Hospital of Soochow University, Suzhou, 215006 Jiangsu People’s Republic of China; 2 Departments of Neurosurgery, The First Affiliated Hospital of Soochow University, Suzhou, 215006 Jiangsu People’s Republic of China; 3 Departments of Thoracic Surgery, The First Affiliated Hospital of Soochow University, Suzhou, 215006 Jiangsu People’s Republic of China

**Keywords:** Brain metastases, Non-small cell lung cancer, Epidermal growth factor receptor, Surgical resection

## Abstract

**Background:**

The standard combination of initial and subsequent treatments of epidermal growth factor receptor (EGFR)-mutated non-small cell lung cancer (NSCLC) patients with solitary brain metastases (BM) remain unclear. Thus, the management options and the progression-free survival (PFS) and the overall survival (OS) of EGFR-mutated NSCLC patients with solitary BM were investigated in the study.

**Methods:**

We retrospectively reviewed the clinical data from NSCLC patients who harbored EGFR mutations and who presented solitary BM at diagnosis in our institute between 2012 and 2014. PFS and OS were evaluated using Kaplan-Meier methods and compared using log-rank tests.

**Results:**

In total, 36 NSCLC patients with solitary BM who harbored EGFR mutations were enrolled in this study. The PFS and OS of these patients was 12.4 and 19.3 months, respectively. Sixteen patients underwent surgical resection of brain and lung lesions followed by EGFR-TKIs treatment, and the median OS was 28.0 months, which was significantly longer than 16.4 months of 14 patients received radiotherapy combined with or followed by EGFR-tyrosine kinase inhibitors (TKIs) and 15.8 months of 6 patients received radiotherapy followed by chemotherapy. The median PFS also showed the same trend in each group (16.1, 10.4, and 9.8 months, respectively).

**Conclusions:**

The survival was extended in the patients receiving surgical resection of brain and lung lesions followed by EGFR-TKIs treatment, and surgery combined with EGFR-TKIs could be a recommended treatment for EGFR mutated NSCLC patients with solitary BM.

## Background

Brain metastases (BM) continue to be one of the most crucial distant metastases in patients with NSCLC, which cause deterioration in quality of life (QOL) and limited life expectancy. Approximately 10–20% NSCLC patients presented BM at diagnosis [1]. Furthermore, if all NSCLC patients had undergone MRI brain at the time of diagnosis, 3% of stage I/II patients and 21% of stage IIIA patients could have been found BM [2].

When NSCLC patients developed BM, the prognosis remains poor with the OS less than 3 months without any treatment [3]. For metastatic disease, systemic therapy is the standard management. However, for NSCLC patients with solitary BM, local treatments of brain and lung lesions may represent useful approaches with a slight improvement in terms of survival, local control, and symptom relief. Three most used treatment options are available for management of brain lesion, including whole brain radiation therapy (WBRT), surgery, and/or stereotactic radiosurgery (SRS). A general consensus has not yet been reached as to which treatment is most effective in such patients. Moreover, up to 40–50% of lung adenocarcinoma patients of East Asian descent harbor EGFR mutations [4]. Previous studies suggested that NSCLC patients with EGFR mutation had higher incidence of BM [5, 6]. Based on the time of discovery of EGFR mutation, these patients may receive EGFR TKIs or chemotherapy as subsequent therapy. There are currently no reported data on which is best comprehensive management approach and PFS as well as OS of EGFR-mutated NSCLC patients with solitary BM. Therefore, in the present study, PFS and OS of EGFR-mutated NSCLC patients with solitary BM treated with surgery or radiation therapy followed by EGFR-TKIs or chemotherapy were investigated.

## Methods

### Patients

Medical data were retrospectively reviewed from NSCLC patients who harbored EGFR mutations and who presented solitary BM at diagnosis in our institute between January 2012 and December 2014. A total of 72 NSCLC patients with solitary BM were evaluated EGFR mutation status in lung biopsy specimens or malignant pleural effusion or bronchoalveolar lavage fluid in our hospital center laboratory. The screening was carried out by polymerase chain reaction (PCR) clamp method. Of these 72 patients, 33 were ineligible because of EGFR wild type and 1 was excluded because of the exon 20T790M mutation, which correlated with resistance to EGFR-TKIs. Twenty patients were detected exon 19 deletion, 17 patients presented exon 21 L858R point mutations, and 1 patient was determined exon 18 G719X point mutation.

Enrolled patients were followed up every 2–3 months and were evaluated based on Response Evaluation Criteria in Solid Tumors (RECIST 1.1). Two of 38 enrolled patients dropped out. Eventually, the information of 36 patients were extracted regarding gender, age, ECOG performance status (PS), recursive partitioning analysis (RPA), graded prognostic assessment (GPA), EGFR mutation types, EGFR-TKI, radiotherapy strategies, date of disease progression and last follow-up, and the outcome of patients from the records.

### Statistical analysis

OS was defined as the time from diagnosis until death or last follow-up. And PFS was defined as the time from the date of initiation of any treatment of surgery or radiotherapy or EGFR-TKIs to the date of documented progression or death. The last follow-up was on 31 December 2016. Categorical variables were compared using Fisher’s exact test. OS and PFS were compared using Log-rank analysis and assessed using the Kaplan-Meier method. The *p* value less than 0.05 was considered statistically significant. Analyses were performed using SPSS 16 software.

## Results

### Patients’ characteristics

**Fig. 1:**
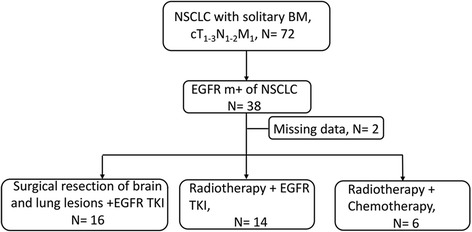
Patient profile

Thirty-six patients with solitary BM who harbored EGFR mutation were included (Fig. 1), and their characteristics are summarized in Table 1. There were 12 (33.3%) males and 24 (66.7%) females with a median age of 58 (39–72) years. The most patients were ECOG PS 0–1 (88.9%). Twenty-seven (75%) patients were assessed at RPA I, and the major GPA score at diagnosis was 2.5–3 (20 patients, 55.6%). Among these 36 patients, 15 were with exon 21 L858R point mutations, 20 with deletion in exon 19, and 1 with other mutation (exon 18 G719X mutation). Twenty-one patients received gefitinib as their EGFR-TKI, while the others (9 cases) received erlotinib. For radiotherapy of brain mass, 16 patients chose SRS and 4 patients chose WBRT. After diagnosis, 16 patients (SUR + TKI group) underwent consecutive surgical resection of brain and lung lesions followed by EGFR-TKIs treatment, among whom 1 patient presented EGFR wild type of brain specimen. Among these patients, 12 patients were diagnosed c&pT_1-3_N_0-1_M_1b_, and 4 patients were diagnosed cT_1-3_N_0-1_M_1b_ before surgery and _P_T_1-3_N_2_M_1b_ from pathologic information after surgery. And 14 patients (RT + TKI group) received radiotherapy combined with or followed by EGFR-TKIs. Moreover, 6 patients (RT + CHE group) received radiotherapy followed by chemotherapy. Among these patients, 2 patients received pemetrexed + carboplatin, 4 patients received pemetrexed + cisplatin. For all aspects of baseline characteristics, there were no statistically significant differences among each group above (*p* > 0.05).

**Table 1:** Patient characteristics

	Total (N)	SUR + TKI (N)	RT + TKI (N)	RT + CHE (N)	*p*
Male/female	12/24	6/10	4/10	2/4	0.884
Age					
Median (range)	58 (39–72)	56.5 (39–68)	57.5 (45–72)	59 (46–63)	0.767
ECOG PS					
0/1/2/3/4	20/12/4/0	12/4/0/0	6/5/3/0	2/3/1/0	0.057
RPA					
I/II/III	27/5	14/2/0	8/3/3	5/0/1	0.114
GPA					
1–2/2.5–3/3.5–4	2/20/14	0/7/9	2/8/4	0/5/1	0.264
EGFR m + type		6/10/0 (lung)			
21/19/others	15/20/1	6/9/0 (brain)	7/6/1	2/4/0	0.934
EGFR-TKI					
Gefitinib/erlotinib	21/19	12/4	9/5	–	0.539
RT					
SRS/WBRT	16/4	–	12/2	4/2	0.355

### Survival analysis

**Fig. 2:**
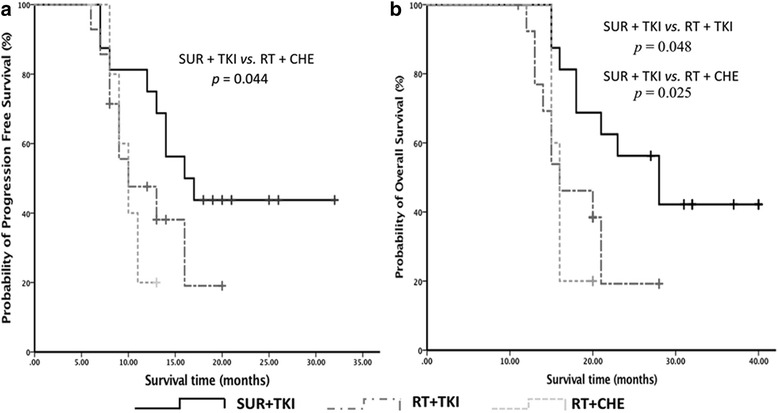
Kaplan-Meier Plot. **a** progression-free survival (PFS). **b** Overall survival (OS)

The median PFS in all 36 patients was 12.4 months (range 5.8–32.1), while the PFS of the patients in SUR + TKI group, RT + TKI group and RT + CHE group were 16.1 months (95%CI 10.1–21.9), 10.4 months (95% CI 3.8–16.2), and 9.8 months (95% CI 7.8–12.1), respectively (Fig. 2a). And the PFS of SUR + TKI group was significantly longer than that of RT + CHE group (*p* = 0.044). No PFS difference was found between SUR + TKI patients and RT + TKI patients (*p* = 0.136), and between RT + TKI patients and RT + CHE patients (*p* = 0.685).

The median survival time of all the patients was 19.3 months (range 11.2–40.3). The OS in SUR + TKI group, RT + TKI group and RT + CHE group were 28.0 months (95% CI 19.2–36.8), 16.4 months (95% CI 10.7–21.3), and 15.8 months (95% CI 15.1–16.9), respectively (Fig. 2b). The patients in SUR + TKI group had prolonged survival than that in RT + TKI group (*p* = 0.048) and than that in RT + CHE group (*p* = 0.025). No OS difference was found between RT + TKI patients and RT + CHE patients (*p* = 0.842).

## Discussion

BM of NSCLC is a frequent complication of NSCLC, which remains a major challenge with relatively limited survival benefits with current therapy strategies. In general, radiotherapy of BM in combination with systemic therapy (chemotherapy and/or molecular target therapy) is the most widely used approach for NSCLC patients with BM. Previous study demonstrated that systemic chemotherapy for NSCLC patients with BM had an OS of 6–8 months [7]. And the median OS of NSCLC patients with BM harboring EGFR mutations treated with EGFR-TKIs is in the range of 15–20 months, which significantly longer than that of EGFR wild-type tumors [8, 9]. In this present study, the median PFS and OS of EGFR mutated NSCLC patients with solitary BM treated with EGFR-TKIs (SUR + TKI group and RT + TKI group) was 13.1 and 20.4 months, respectively, which is similar to previous researches. To our knowledge, this is the first study of compared management options and calculated the PFS and OS of EGFR mutated NSCLC patients with solitary BM.

Notably, for patients with solitary BM, the OS may be different from that with multiple metastases, and the local treatment may be the first option. A study showed that 74.6% NSCLC patients with solitary BM survived more than 1 year, and single brain metastasis is commonly considered to be a favorable prognostic factor [10]. It was well accepted that the standard treatment of NSCLC patients with solitary BM was surgical resection of solitary brain lesions and with WBRT and/or SRS. Tissue diagnosis is still the gold standard as pathological diagnosis. As early as 1990, a study showed that 6 of 59 patients thought to BM actually had an abscess, benign tumor or another primary tumor [11]. This case indicates that some brain lesions thought to be metastases of NSCLC might be misdiagnosed and received invalid treatments. Furthermore, a case report of a patient whose primary lesion harbored an exon 20T790M mutation, while the BM specimen was negative for the mutation [12]. And in this present study, 1 patient presented deletion in exon 19 of lung lesion and EGFR wild type of brain lesion. These all indicates that the gene performance of BM may differ from that of the primary lesion, and surgery of BM will help not only to remove of cranial lesion but also to allow exact tissue and gene diagnosis and indicate the best management approach. Additionally, in this study, patients receiving surgical resection of brain and lung lesions followed by EGFR-TKIs treatment gained significantly longer survival than radiotherapy combined with EGFR-TKIs or radiotherapy in combination with chemotherapy. These data suggest that surgical intervention combined with EGFR-TKIs is beneficial for EGFR mutated NSCLC patients with solitary BM.

Another explanation for the survival benefit of surgical intervention combined with EGFR-TKIs group is that patients in this group underwent surgeries of lung tumor. Several series have previously reported encouraging survival outcomes after lung surgery in patients with synchronous BM [13, 14], and our findings supported this conclusion. Moreover, EGFR-TKIs treatment is another possible beneficial factor, which is the standard management as first line of advanced NSCLC patients harboring EGFR mutations. Recently, an increasing interest has been presented in the role of EGFR-TKIs in the treatment of BM from NSCLC. Evidences suggest that EGFR-TKIs can cross the blood-brain barrier (BBB) [15], while most chemotherapeutic agents cannot cross BBB. However, for the patients who underwent surgery of both brain and lung lesions, whether EGFR-TKIs are suitable as adjuvant treatment remains unclear, while currently there was no strong evidence of EGFR-TKIs as adjuvant treatment of local NSCLC. In this study, patients with surgery of both brain and lung lesions followed by EGFR-TKIs had prolonged survival, which supported that EGFR-TKIs are recommended as adjuvant treatment of EGFR mutated NSCLC patients with solitary BM.

In this study, WBRT was not used as initial therapy followed surgery of BM. When 10 patients presented recurrence of brain disease several months later, they received WBRT at that time. A retrospective study demonstrated that WBRT of patients had deleterious results [16]. Moreover, another study showed that compared to the WBRT group, patients in the observation group had better mean scores in physical, role, and cognitive functioning at 8 weeks, and thus the WBRT can be delayed [17].

The most remarkable limitation of this study is that the study conducted at a single institution and the number of patients is relatively small. Secondly, subsequent therapy and/or further therapy could not be fully evaluated due to limited patients sample size. However, as the first study investigating the role of management options and the PFS and OS of EGFR mutated NSCLC patients with solitary BM, the findings are clinically meaningful.

## Conclusions

The survival was prolonged in the patients receiving surgical resection of brain and lung lesions followed by EGFR-TKIs treatment, in comparison with that received radiotherapy of BM combined with EGFR-TKIs or radiotherapy of BM followed by chemotherapy. Surgery combined with EGFR-TKIs could be a recommended treatment for EGFR mutated NSCLC patients with solitary BM. In the future, larger and prospective studies will be needed to confirm these results.

## Abbreviations

BM: brain metastases
ECOG: Eastern Cooperative Oncology Group
EGFR: Epidermal growth factor receptor
GPA: Graded prognostic assessment
m +: Mutation-positive
NSCLC: Non-small cell lung cancer
OS: Overall survival
PCR: Polymerase chain reaction
PFS: Progression free survival
PS: Performance status
QOL: Quality of life
RPA: Recursive partitioning analysis
RT: Radiotherapy
SRS: Stereotactic radiosurgery
SUR: Surgical resection of brain and lung lesions
TKIs: Tyrosine kinase inhibitors
WBRT: Whole brain radiation therapy
